# Heart disease in pregnancy—clinical pattern and prevalence: initial data from the first cardio-maternal unit in Iraq

**DOI:** 10.1186/s13104-019-4523-6

**Published:** 2019-08-07

**Authors:** Hasan Ali Farhan, Israa Fadhil Yaseen

**Affiliations:** 1 Scientific Council of Cardiology, Iraqi Board for Medical Specializations, Baghdad, Iraq; 2 Baghdad Heart Center, Baghdad Teaching Hospital, Medical City, Baghdad, Iraq

**Keywords:** Pregnancy, Heart disease, Clinical pattern, Prevalence

## Abstract

**Objectives:**

The purpose of this study to determine the clinical pattern and prevalence of heart disease in pregnancy at the first established cardio-maternal unit in Iraq over the last 4 years; since January 2015 till May 2019. Data are presented as number and percentage.

**Results:**

A total of 252 pregnant women presented to cardio-maternal unit included in this study. According to the collected data, among the main diagnosis of heart disease during pregnancy was valvular heart disease 34.1%, followed by congenital heart disease 30.5%, cardiomyopathy 29.8%, pulmonary hypertension 4%, and ischemic heart disease 1.6%. Among subtypes of the main heart diseases in pregnant women, the most clinical pattern was: the prosthetic heart valve (26.7%) in valvular heart disease, both atrial septal defect and ventricular septal defect (35%) in congenital heart disease, and peripartum cardiomyopathy (76%) among cardiomyopathies.

## Introduction

Cardiovascular disease (CVD) in women is associated with 4% complications during their pregnancy and is the most frequent leading cause of maternal mortality reaching up to 15% [1–4]. After 2010, the rate of maternal mortality mainly in emerging countries began to decline, despite increasing numbers of high-risk patients [2]. This decline may be related to improvement in medical service; which generally includes cardiologist, obstetrician, and anesthetist, following European Society of Cardiology guidelines for management of CVD during pregnancy, and registration of patients’ data which is improving significantly in the emerging countries as it was shown while comparing data from PREG1 and PREG2 cohorts (21.3% vs 45.3%); PREG1 and PREG2 cohort data were collected from the main international “Registry Of Pregnancy And Cardiac disease” which was established by the EURObservational Research Programme of the European Society of Cardiology to include large number of pregnant women with CVD from the real world daily practice to identify the outcomes of these patients and in order to improve their outcomes as an ultimate goal [2, 5, 6]. Cardiovascular registries have an essential role in the prevention, diagnosis and treatment of CVD in the real world practice consequently, provide an evidence based of the clinically important outcomes and they are a key tool for enhancing and raising the standard of the health and patients care [7, 8]. In this article, we will demonstrate for the first time the clinical pattern and prevalence of heart disease (HD) in pregnant women presented to the first established cardio-maternal unit in Iraq over 4 years, this will encourage to spread the registration nationally and internationally leading to improve prevention, diagnosis and management of HD during pregnancy.

## Main text

### Methods

A total of 252 pregnant women with heart disease who presented to cardio-maternal unit/Baghdad Heart Center/Baghdad Teaching Hospital/Medical City between January 2015 and May 2019 were included in this study. Patients were referred to cardio-maternal unit either with a known case of heart disease or with symptoms requiring cardiac investigation (electrocardiogram or echocardiogram) to confirm the diagnosis. Patients were referred from in-hospital patients, outpatient clinic at the hospital, private clinics outside the hospital, or from other cities because of the complexity of cases that can not be managed and followed up in their cities. The study was performed in accordance with the ethical standards of the Declaration of Helsinki.

### Results

**Fig. 1:**
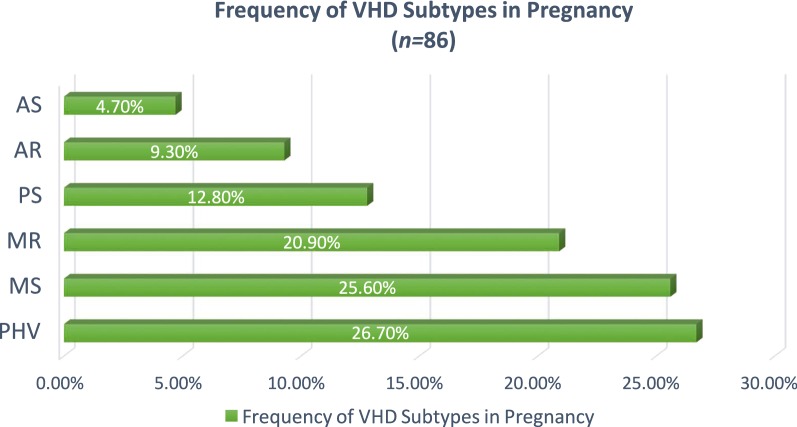
Frequency of valvular heart disease subtypes in pregnancy. *AR* aortic regurgitation, *AS* aortic stenosis, *MR* mitral regurgitation, *MS* mitral stenosis, *PHV* prosthetic heart valve, *PS* pulmonary stenosis, *VHD* valvular heart disease

According to the collected data (252 patients), among the main diagnosis of HD during pregnancy was valvular heart disease (VHD) 34.1%, followed by congenital heart disease (CHD) 30.5%, cardiomyopathy 29.8%, pulmonary hypertension 4%, and ischemic heart disease 1.6%; (Table 1). Among subtypes of the main heart diseases in pregnant women, the most clinical pattern was: the prosthetic heart valve (26.7%) in VHD (Fig. 1), both atrial septal defect and ventricular septal defect (35%) in CHD, and peripartum cardiomyopathy (PPCM) (76%) among cardiomyopathies.

**Table 1:** Clinical pattern and prevalence of heart disease in pregnancy at cardio-maternal clinic

Main diagnosis	Total no. (%)	Subtypes	Subtypes no. (%)
VHD	86 (34.1%)	PHV	23 (26.7%)
		MS	22 (25.6%)
		MR	18 (20.9%)
		PS	11 (12.8%)
		AR	8 (9.3%)
		AS	4 (4.7%)
CHD	77 (30.5%)	ASD	27 (35%)
		VSD	27 (35%)
		TOF	8 (10.4%)
		Miscellaneous	8 (10.4%)
		PDA	7 (9%)
CMP	75 (29.8%)	PPCM	57 (76%)
		DCM	11 (14.7%)
		HOCM	4 (5.3%)
		Non-compaction	3 (4%)
PHT	10 (4%)		
IHD	4 (1.6%)		
	252 (100%)		

*AR* aortic regurgitation, *AS* aortic stenosis, *ASD* atrial septal defect, *CHD* congenital heart disease, *CMP* cardiomyopathy, *DCM* dilated cardiomyopathy, *HOCM* hypertrophic cardiomyopathy, *IHD* ischemic heart disease, *MR* mitral regurgitation, MS mitral stenosis, *PDA* patent ductus artriosus, *PHT* pulmonary hypertension, *PHV* prosthetic heart valve, *PPCM* peripartum cardiomyopathy, *PS* pulmonary stenosis, *TOF* tetralogy of Fallot, *VHD* valvular heart disease, *VSD* ventricular septal defect

### Discussion

This study is showing the clinical pattern of HD in pregnancy among Iraqi patients at the first established cardio-maternal unit over the last 4 years, revealing that VHD as the main clinical pattern among pregnant patients and this is agreed with the current available and updated evidence that documented rheumatic VHD as the most common HD in pregnancy in emerging countries, which complicated 50–90% of HD in pregnancy and associated with the higher rate of mortality comparing with other HD [3, 4, 6, 9–12]. Prosthetic heart valve and mitral stenosis (MS) were predominate among pregnant women with VHD, and it is very important to focus on these subtypes of HD as prosthetic heart valve and MS are associated with high maternal and fetal complications, in addition, MS is considered as the most common leading cause for maternal mortality in patients with VHD in emerging countries especially postpartum mortality because it is poorly tolerated during pregnancy [3, 4, 9, 11–13]. Documentation the prevalence of VHD during pregnancy is essential to improve the standard of care of this condition especially in the emerging countries because despite the high burden of VHD particularly rheumatic heart disease (RHD) in these countries, still there is a limited data and studies on this topic, moreover, it will help in reducing the financial burden as the surgical intervention of RHD presents a high financial burden even in higher income countries [3, 13, 14]. For this reason, the World Heart Federation Working Group on rheumatic fever and RHD published a paper declaring a commitment to fight these diseases and to draw a detailed operational plan in order to control this burden of HD [12]. The second most prevalent HD in pregnancy was CHD. The prevalence of pregnant women with CHD is difficult to determine due to the methodology used in the reported studies [10]. The rate of deliveries of pregnant women with CHD rises by 34.9% over 10-years period (1998–2007) which is explained by the advance diagnostic, medical and surgical management and care leading to improve the survival of more than 90% patients with CHD, reaching the reproductive age even those with more complex CHD [15–18]. However, pregnant women with CHD are more prone to develop cardiovascular and adverse obstetric and fetal complications, even the simple CHD can hold high pregnancy risk if their condition is associated with other comorbid disease [15, 17, 18]. Fortunately, most of these complications can be managed and the rate of mortality is rare [10, 16]. Cardiomyopathy; mainly PPCM, followed VHD and CHD in this study. Incidence of PPCM varies depending on geographical regions [19]. It is a serious condition especially one-month post diagnosis and associated with higher rate of major cardiovascular events reaching (46%) comparing with dilated cardiomyopathy (39%), therefore, requiring intensive and multidisciplinary management to improve outcomes [5, 20, 21]. In Summary, study and precise registration of pregnant women with HD is an important tool to improve the standard of care for those patients, reducing morbidity and mortality, and to reduce the health financial burden. Moreover, establishing a cardio-maternal unit with a multidisciplinary team is the cornerstone for optimizing care and management of HD in pregnancy.

### Conclusion

This study showed for the first time the clinical pattern and prevalence of heart disease in pregnancy among Iraqi patients who were presenting to the first cardio-maternal unit in the country. The results demonstrated valvular heart disease as the main type of heart disease among the cohort patients followed by congenital heart disease and cardiomyopathy; particularly peripartum cardiomyopathy. Current data regarding heart disease during pregnancy is very limited, therefore, precise registration of such data is very essential for the future prevention and management of those patients and consequently to improve their outcomes by a multidisciplinary team in a specialized unit.

## Limitation

Data include pregnant women with heart disease who were referred to our cardio-maternal unit does not reflect the real prevalence in our community.

## Data Availability

The datasets used and/or analyzed during the current study are available from the corresponding author on reasonable request.

## Abbreviations

CVD: cardiovascular disease
HD: heart disease
VHD: valvular heart disease
CHD: congenital heart disease
MS: mitral stenosis
RHD: rheumatic heart disease
